# A first look at consistency of documentation across care settings during emergency transitions of long-term care residents

**DOI:** 10.1186/s12877-023-03731-6

**Published:** 2023-01-11

**Authors:** Kaitlyn Tate, Rachel Ma, R. Colin Reid, Patrick McLane, Jen Waywitka, Garnet E. Cummings, Greta G. Cummings

**Affiliations:** 1 Faculty of Nursing, University of Alberta, Edmonton, AB Canada; 2 University of British Columbia - Okanagan Campus, Kelowna, BC Canada; 3 School of Health and Exercise Sciences, University of British Columbia - Okanagan Campus, Kelowna, BC Canada; 4 Emergency Strategic Clinical NetworkTM, Alberta Health Services (AHS), Edmonton, AB Canada; 5 Department of Emergency Medicine, Faculty of Medicine & Dentistry, University of Alberta, Edmonton, AB Canada; 6 Alberta Health Services, Edmonton, Canada

**Keywords:** Transitions, Communication, Nursing homes, Long-term care, Emergency department, Documentation

## Abstract

**Background:**

Documentation during resident transitions from long-term care (LTC) to the emergency department (ED) can be inconsistent, leading to inappropriate care. Inconsistent documentation can lead to undertreatment, inefficiencies and adverse patient outcomes. Many individuals residing in LTC have some form of cognitive impairment and may not be able to advocate for themselves, making accurate and consistent documentation vital to ensuring they receive safe care. We examined documentation consistency related to reason for transfer across care settings during these transitions.

**Methods:**

We included residents of LTC aged 65 or over who experienced an emergency transition from LTC to the ED via emergency medical services. We used a standardized and pilot-tested tracking tool to collect resident chart/patient record data. We collected data from 38 participating LTC facilities to two participating EDs in Western Canadian provinces. Using qualitative directed content analysis, we categorized documentation from LTC to the ED by sufficiency and clinical consistency.

**Results:**

We included 591 eligible transitions in this analysis. Documentation was coded as *consistent*, *inconsistent*, or *ambiguous*. We identified the most common reasons for transition for consistent cases (falls), ambiguous cases (sudden change in condition) and inconsistent cases (falls). Among inconsistent cases, three subcategories were identified: *insufficient reporting*, potential *progression of a condition* during transition and *unclear* reasons for inconsistency.

**Conclusions:**

Shared continuing education on documentation across care settings should result in documentation supports geriatric emergency care; on-the-job training needs to support reporting of specific signs and symptoms that warrant an emergent response, and discourage the use of vague descriptors.

## Background

Documentation of resident conditions during transitions from long-term care (LTC) to the emergency department (ED) can be incomplete or nonspecific [1–3]. Inconsistent or nonspecific reporting of symptoms resulting from changes in health condition can put older adults (≥ 65 years of age) at risk of inappropriate and/or insufficient care [3–5]. Having various health care professionals involved in a transition that occurs across numerous care settings (LTC, emergency medical services [EMS], the ED and back) renders continuity of care and effective communication challenging propositions. Information continuity, where documentation of resident or patient condition is consistent across care settings, is critical to ensure residents receive coherent and effective management of their health condition [6, 7]. Communication difficulties are exacerbated as older adults from LTC settings often have some level of cognitive impairment and may not be able to clearly communicate their symptoms and care needs [8, 9]. The potential downstream effects of poor or insufficient documentation in these instances could be far-reaching when we consider that cognitive impairment leads to a higher risk of negative outcomes in various healthcare settings, particularly hospitals [10]. Adding to this, healthcare professionals (HCPs) caring for older adults during these transitions often do not have adequate training or education in geriatrics, and report witnessing ageism during emergency care for older adults through the rationing or delay of care [11, 12]. As older adults may present with unclear or atypical symptoms for serious changes in health condition, precise and informative documentation is critical to direct effective care that combats the potential effects of discrimination against older adults who may be unable to advocate for themselves [13, 14].

Sub-standard quality of care is a feature during each stage of the transition including at the LTC facility, [15] during emergency transport (i.e. via ambulance) and in the ED [16, 17]. Both residents and their families report unmet needs during these transitions, related to both physical health (e.g., dehydration, being cold and in pain, requiring a toilet and/or requiring help due to physical impairment) and mental or emotional health (e.g., being in a state of confusion, being frightened or anxious, not understanding medical language) [18]. Consistent and accurate documentation during resident transitions from LTC to the ED, especially pertaining to the reason for emergency transition, are vital in determining and providing timely, sufficient and appropriate care to address the presenting health concern (e.g., delirium) and to support quality care during the transition [4, 5, 19]. Discrepancies and insufficient reporting related to reasons for transfer to the ED have been identified as issues [20, 39]. However, the nature of those discrepancies has not been explored. Understanding these discrepancies could result in findings that support targeted research and intervention development to improve documentation practices.

This paper reports the results of an analysis of data from the Older Persons’ Transitions in Care (OPTIC) study. The overarching OPTIC study was an observational census study funded by the Canadian Institutes of Health Research (CIHR), [21] during which we analyzed all LTC—ED transfers in two Canadian cities over a one-year period and developed a tool to measure transition success [22]. In this paper, we analyzed documentation of the resident’s condition in LTC, EMS (emergency transport via ambulance) and the ED, during transition to the ED from LTC. Specifically, the reason for transition reported at each setting was examined. In doing so, our objectives were to:

1. examine and compare the nature of inconsistent and consistent documentation during emergency transitions from LTC to the ED, and
2. determine if any reasons for emergency transitions were more common among transfers exhibiting inconsistent documentation.

## Methods

### Design

OPTIC was an observational census study of transitions from LTC to ED and back via EMS using a standardized data collection form, the Transition Tracking Tool (T3) [19]. The T3 was developed by researchers and knowledge users and pilot tested on an initial 54 transfers [22, 23]. For the purposes of this paper, we conducted resident chart/patient record review and analysed documentation using directed content analysis [24–26].

#### Sample

We included residents of LTC aged 65 or over at the time of the study who were transferred to ED by EMS on an emergency basis. We did not exclude participants based on disposition from the ED (i.e., they may have been discharged directly back to LTC, been admitted, died while in hospital or been discharged to a different facility). In this analysis, we used transitions, rather than residents, as our unit of analysis (i.e., one resident may have experienced multiple transitions).

#### Setting

A combined thirty-eight LTC facilities and two EDs in Kelowna, British Columbia (BC), and Edmonton, Alberta (AB) took part in the study. All LTC facilities in Kelowna participated (13 facilities), as well as 25 of 37 invited LTC facilities in Edmonton. Two ambulance services (the only services for each city) were invited to participate. Alberta EMS participated; however, BC Ambulance Services chose not to participate in the study. Kelowna ED participated in this study, and one tertiary teaching ED was selected for the study in Edmonton [23]. LTC characteristics are reported in the Reid et al., pilot study preceding this study [22].

#### Ethic

Ethics approval was granted by the University Alberta Health Research Ethics Board (HREB B: Pro00010666; Pro00017240) for AB and Interior Health Research Office and Research Ethics (UBCO BREB: 2010–017) as well as the University of British Columbia Okanagan Behavioural Research Ethics Board (UBCO BREB: H10-00,127) for BC. A waiver of consent was obtained for individuals participating in this study. This waiver was obtained due to the high number of residents with cognitive impairment, and the impracticality of reaching substitute decision makers in a timely manner during an emergency transfer. The regional health authority in BC issued a waiver of consent for transferred residents from nine of the 12 LTC facilities participating in the study. The remaining three LTC facilities in BC required that residents or their family members provide consent prior to research staff accessing their nursing home care records, though hospital records were granted a waiver of consent for these residents. In AB, a waiver of consent was granted by all participating LTC facilities. This waiver of consent was approved by the ethics boards of the University of Alberta, the Interior Health Research Office, and the University of British Columbia Okanagan, per the ethics applications noted above. This research was conducted in accordance with the Declaration of Helsinki.

#### Measures

The T3 [20, 21] allowed for the collection of data on individual LTC residents experiencing transitions. The following case information was used in data analysis: resident comorbidities, primary trigger event leading to transfer from LTC, EMS reported chief complaint, ED chief complaint, ED diagnosis, Cognitive Performance Scale score and patient acuity in the ED measured using the Canadian Triage and Acuity Scale (CTAS) score [27]. This 5-point scoring system is applied at triage by trained ED nurses in both provinces. The possible scores (and recommended time frames for care) are: 1: resuscitation (immediate); 2: emergent (< 15 min); 3: urgent (< 30 min); 4: semi-urgent (< 60 min); 5: non-urgent (< 120 min) [26].

#### Data collection procedure

Trained research assistants (RAs) collected data from health records in person at each care setting from July 2011 to July 2012. Using tablets, they uploaded data to a secure server created and maintained by Nooro Online Research (https://nooro.com/). In many cases data were unstructured “open” text for presenting complaints, procedures and diagnoses that did not clearly match categories established by clinician researchers [20].

#### Data analysis

We employed directed qualitative content analysis to determine whether the chain of documentation from LTC to the ED and back was sufficient and clinically consistent [24–26]. Transition documentation was defined as *consistent* if the LTC reason for transfer aligned with expected symptomology documented by EMS and ED, or if signs and symptoms clearly described an event that could logically be the cause of those same signs and symptoms (e.g., if reason for transfer was reported in LTC as a fall, and hip pain was reported subsequently, this was considered consistent documentation). Transition documentation was defined as *inconsistent* when symptomatology, trigger events and diagnoses did not align in a clinically intuitive and sequential manner across the transition. Content analysis is an appropriate approach for analyzing information obtained through chart review [26]. Directed content analysis infers meaning by counting and comparing codes within predetermined content categories to describe a phenomenon, in this case the documentation by HCPs during emergency transitions of LTC residents [25]. Directed content analysis is appropriate when existing theory, research or logic models can help direct the coding scheme for a more deductive approach (e.g., our understanding of how sepsis might develop provides a framework to support our decisions regarding whether the reported symptoms align with the potential progression of the condition and diagnosis) [24, 25]. This method relies on content experts to validate the use of particular categories and understandings, in this case logic models related to emergency transitions of older adults. Three co-authors of this article are registered nurses (RNs), with clinical and research experience in emergency departments and long-term care settings (KT, JW, GGC). One co-author is an emergency medicine physician (GEC) and another co-author is an emergency medical responder, with experience in emergency medical services (RM).

**Fig. 1:**
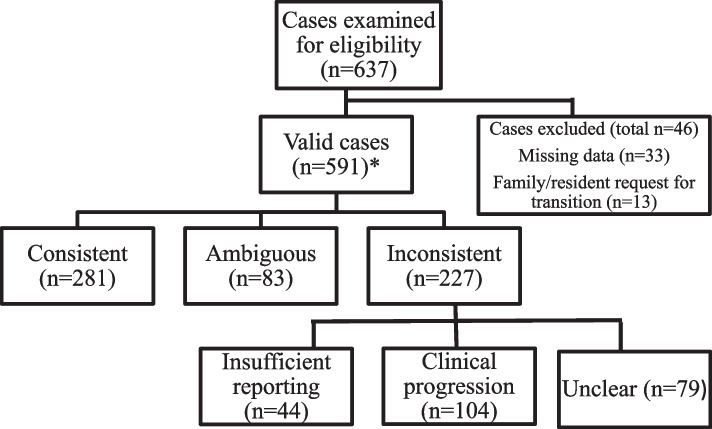
Flow Diagram of Case Inclusion and Coding Categories to Describe Documentation Related to Reason for Transfer

Three research team members (KT, JW, RM) independently analysed the case data from each transition. We specified our unit of analysis and developed a formative categorization framework by inductively coding the transition documentation as consistent or inconsistent [24]. We defined our categories and subcategories, selected anchor or exemplar cases for each category and determined coding rules using an iterative process in which we scheduled team consensus meetings after pretesting the coding framework and during the main analysis [24, 25]. Specifically, cases were considered consistent if, in documentation, HCPs: 1) reported the same terms for signs or symptoms, 2) used different terms that reasonably referred to the same sign or symptom (e.g., suprapubic pain and lower abdominal pain), or 3) included the condition or event of concern and the sign or symptom of that condition or event (e.g., fall and hip pain, gastrointestinal bleed and bleeding). Similar cases were grouped into categories. Cases for which independent coders found achieving consensus challenging were presented to the study principal investigator for review (also a RN with substantive and clinical expertise in critical and older persons’ care). Categories of cases, which included the list of individually coded cases, were presented to the principal investigator for additional review and subsequent consensus discussion. Discrepancies in independent coding were resolved through consensus discussion. Team meetings were held throughout the analysis process and following the completion of final coding to discuss findings and reach consensus. During coding, a new category emerged which we labeled *ambiguous* in documentation. These were cases in which the primary reason for transfer was reported as ‘sudden change in physical condition’ and the EMS and ED chief complaints were consistent (e.g., both the EMS and ED chief complaint were ‘general malaise’ or ‘stroke’). We did not identify these as *consistent* as the reason for transition from LTC was deemed by research team members as insufficient for providing clinical direction, but not technically contrary to any subsequent documentation during the transition. A flow diagram outlining the coding categories can be seen in Fig. 1.

Data went unrecorded when RAs could not access the chart. Cases with missing data from any care setting for this reason were excluded because we could not determine whether documentation across the transition was reported or clinically consistent. We also excluded cases when the reason for transfer was resident/family request, as no clinical problem was recorded. Cases in which nothing was written by a HCP were marked as ‘Not recorded’ by research assistants and were included in analysis as valid cases. We also provide descriptive results, specifically frequencies and percentages, when describing the results of each content category.

## Results

### Descriptive results of sample

In OPTIC, a total of 637 LTC-ED-LTC (AB = 398, BC = 239) transitions of 524 residents was tracked over a 12-month period, with data collected from each care setting. In 33 transitions, data from at least one care setting were missing because research staff were not able to obtain access to resident charts. In 13 cases, resident or family request was documented as the reason for transfer. We included 591 transitions in this analysis. All residents involved in these transitions had at least one pre-existing diagnosis, the most common being hypertension (61.7%), dementia (59.9%) and arthritis (40.8%). For all included 591 transitions, the majority of residents were male (*n* = 360, 61.1%) and the mean age was 84.3 years (SD = 7.7). Similarly, for transitions with inconsistent documentation, the majority of residents were male (55.1%) and the mean age was 84.0 years (SD = 8.3). Descriptive results for transitions with consistent documentation reflected similar patterns. Descriptive results across content categories and for the total number of transitions examined in this study are also presented for transitions (see Table 1).

**Table 1:** Descriptive statistics of resident characteristics during transitions from long-term care to the emergency department

Variable (Valid N)		Transitions with consistent documentation (*n* = 281)	Transitions with ambiguous documentation (*n* = 83)	Transition with inconsistent documentation (*N* = 227)	Total (Valid *N* = 591)
Sex [N(%)]	Female	98 (34.9)	30 (36.1)	102 (44.9)	230 (38.9)
	Male	183 (65.1)	53 (63.9)	125 (55.1)	361 (61.1)
Age [mean(SD)]	Age in years	84.84 (7.1)	83.42 (8.1)	84.03 (8.2)	84.33 (7.7)
	Missing	1	0	0	1
Most frequent pre-existing diagnoses [Check all that apply, N(%)]	Hypertension	171 (60.9)	49 (59.0)	145 (63.8)	365 (61.7)
	Dementia	162 (57.7)	59 (71.1)	133 (58.6)	354 (59.9)
	Arthritis	109 (38.8)	34 (41.0)	98 (43.6)	241 (40.8)
	Mental Health/Psychiatric Issue	120 (42.7)	30 (36.1)	83 (36.9)	233 (39.6)
	Cardiovascular Disease	80 (28.5)	26 (31.3)	86 (38.2)	192 (32.5)
	Stroke	86 (30.6)	29 (34.9)	71 (31.6)	186 (31.5)
	Osteoporosis	108 (38.4)	16 (5.3)	64 (28.2)	188 (31.8)
	Diabetes	67 (23.8)	30 (36.1)	67 (29.8)	164 (27.7)
	Thyroid Disease	90 (32.0)	19 (22.9)	56 (24.9)	165 (27.9)
	Vision Disease	83 (29.5)	25 (30.1)	55 (24.2)	163 (27.6)
Cognitive Performance Scale Score [N(%)]	0 = decision-making intact	23 (8.2)	8 (10.0)	18 (8.6)	49 (8.7)
	1 = decision-making borderline intact	33 (11.7)	10 (12.5)	19 (9.1)	62 (11.0)
	2 = decision-making mild impairment	65 (23.1)	15 (18.8)	43 (20.6)	123 (21.8)
	3 = decision-making moderate impairment	86 (30.6)	33 (41.3)	74 (35.4)	193 (34.2)
	4 = decision-making moderately severe impairment	27 (9.6)	5 (6.3)	20 (9.6)	52 (9.2)
	5 = decision-making severe impairment	25 (8.9)	4 (5.0)	22 (10.5)	51 (9.0)
	6 = decision-making very severe impairment	16 (5.7)	5 (6.3)	13 (6.2)	34 (6.0)
	Missing	6	3	16	25
Advance Directive [N(%)]	Yes	142 (50.5)	50 (61.0)	154 (67.8)	386 (66.7)
	No	139 (49.5)	32 (39.0)	72 (31.7)	193 (33.3)
	Missing	0	0	1	1
Canadian Triage and Acuity Scale Score in Emergency [N(%)]	1 = Resuscitation	12 (4.3)	6 (7.3)	5 (2.2)	23 (3.9)
	2 = Emergent	54 (19.5)	33 (40.2)	90 (40.2)	177 (30.4)
	3 = Urgent	145 (52.3)	39 (47.6)	105 (46.9)	289 (49.6)
	4 = Less urgent	63 (22.7)	3 (3.7)	21 (9.4)	87 (14.9)
	5 = Non-urgent	3 (1.1)	1 (1.2)	3 (1.3)	7 (1.2)
	Missing	4	1	1	6
	^a^Missing refers to cases when the study research assistant was not able to access the data source				

^a^105 residents experienced repeat transitions to the ED within the study period, ranging from 2 (n = 89) to 5 transitions (n = 2)

### Directed content analysis results

Of 591 transitions, 281 (47.5%) were *consistent* in the chain of documentation that followed the reason for transfer, across care settings. Eighty-three (14%) transitions were identified as *ambiguous* in documentation that followed the reason for transfer*.* In 227 transitions (38.4%), documentation was assessed as inconsistent, and were subcategorized further into: 1) *insufficient reporting,* 2) *dynamic disease condition or progression, and* 3) *unclear reason for inconsistent documentation.* Case examples for each subcategory are provided in Table 2.

**Table 2:** Case examples for each category

Category	***Trigger events that lead to transfer***	***EMS chief complaint***	***ED chief complaint***	***Diagnosis in ED***
Consistent (*n* = 281):				
	Fall(s)	Hip/Pelvis/Back Pain	Hip/Pelvis/Back Pain	Hip/pelvis fracture
	Short of Breath	Short of Breath	Short of Breath	Heart failure
	Leg Pain/Cramps/Swelling	Leg Pain/Cramps/Swelling	Leg Pain/Cramps/Swelling	Cellulitis
Ambiguous (*n* = 83):				
	Sudden Change in Physical Condition	Change in Level of Consciousness	Change in Level of Consciousness	Pneumonia
	Sudden Change in Physical Condition	General Malaise/Weakness	General Malaise/Weakness	Urinary Related Disease
	Sudden Change in Physical Condition	Gastro-intestinal (GI) Bleed	GI Bleed	GI Bleed
Insufficient reporting (*n* = 44):				
	Not Recorded/No Data Available/Illegible	Head/Neck trauma	Head/Neck Trauma	Head Contusion/Laceration
	Not Recorded/No Data Available/Illegible	General Malaise/Weakness	General Malaise/Weakness	General Malaise/Weakness
	Chest Pain	Not Recorded/No Data Available/Illegible	Chest Pain	Cardiac Arrythmia
	Fever	Transfer	Urinary Related Disease	Urinary Related Disease
Disease progression (*n* = 104):				
	Fall(s)	Change in Level of Consciousness	Confusion/Delirium	Stroke
	Cough with Congestion	Short of Breath	Cough with Congestion	Respiratory Related Illness
	GI Bleed	Short of Breath	Short of Breath	Cardiac/Respiratory Failure
	Urinary Related Disease	Change in Level of Consciousness	Cardiac Arrythmia	Urinary Related Disease
Unclear (*n* = 79):				
	Fall(s)	Urinary Related Disease	Constipation/Bloating/Abdominal Pain	Constipation/Bloating/Abdominal Pain
	Change in Behaviour (agitation, aggression)	Mental Health Issues/Psychiatric Issues	General Malaise/Weakness	Dementia
	Short of Breath	Bleeding	Urinary Related Disease	Sepsis
	Constipation/Bloating/Abdominal Pain	Musculoskeletal Pain	Nausea/Vomiting/Diarrhea	Fall(s)
	Fall(s)	Swallowing	Medical Device Problem	Pulmonary Embolism

The most common reasons for transition among *consistent* cases were falls (n = 124) and shortness of breath (n = 46). The reasons for transfer for consistent and inconsistent cases can be viewed in Table 3. The reason for transition among all 83 cases of *ambiguous* documentation was ‘sudden change in physical condition’.

**Table 3:** Reason for Transition Among Consistent (n = 281) and Inconsistent Cases (n = 227)

Reason for transfer	# of cases consistent cases [N(%)]	# of cases inconsistent cases [N(%)]
Falls/post fall injury	126 (44.8)	41 (18.1)
Shortness of breath	46 (16.4)	27 (9.6)
Skin changes/wounds	12 (4.3)	3 (1.3)
Urinary related disease/catheter issues	10 (3.6)	7 (3.1)
Constipation/bloating/abdominal pain	9 (3.2)	7 (3.1)
Leg pain/cramps/swelling	9 (3.2)	3 (1.3)
Chest pain	8 (2.8)	8 (3.5)
Gastrointestinal bleed	7 (2.5)	7 (3.1)
Nausea/vomiting/diarrhea	7 (2.5)	8 (3.5)
Epistaxis	7 (2.5)	0
Gastrointestinal tube	7 (2.5)	2 (0.9)
Change in level of consciousness	6 (2.1)	0
Seizures/tremors	4 (1.4)	1 (0.4)
Hip/pelvis/back pain	4 (1.4)	1 (0.4)
General malaise/weakness	3 (1.1)	11 (4.8)
Sudden change in physical condition	2 (0.7)	41 (18.1)
Change in behaviour (agitation, aggression)	2 (0.7)	6 (2.6)
Fracture	2 (0.7)	0
Contusions/lacerations	2 (0.7)	1 (0.4)
Stroke	2 (0.7)	0
Low blood sugar	1 (0.4)	0
Aspiration pneumonia	1 (0.4)	5 (2.2)
Swallowing	1 (0.4)	3 (1.3)
Cough with congestion	1 (0.4)	10 (4.4)
Tracheostomy tube	1 (0.4)	1 (0.4)
Stroke	1 (0.4)	0
Nothing reported	0	14 (6.2)
Genital issues	0	3 (1.3)
Edema	0	2 (0.9)
Bleeding	0	1 (0.4)
Cardiac arrythmia	0	1 (0.4)
Renal failure	0	1 (0.4)
Fever	0	1 (0.4)
Other	0	1 (0.4)

We did not include ambiguous cases in this table as ‘sudden change in physical condition’ was the reported reason for transfer in all of these cases (*n* = 83)

In 227 transitions (38.2%), documentation was assessed as *inconsistent* with the reason for transfer (see Table 3). In 44 of 227 transitions (19.3%), *inconsistent* documentation was based on *insufficient reporting* alone. This meant that, although the research assistant could access the chart, nothing was reported as a clinical reason for transfer. In 27 transitions (61.4%) the chief resident complaint during EMS transfer was not reported, or the reason reported was only ‘transfer’ or ‘no patient care record’, in 13 of these transitions (29.5%) the primary reason for transfer from LTC was not reported, and in two transitions (4.5%) no reason for transfer or ineligible data were reported from the ED.

In 104 of 227 transitions (45.6%), inconsistent documentation reflected a likely progression in the acute condition afflicting the resident, or different manifestations of the same condition. For example, a transition may start with cough with congestion in LTC followed by shortness of breath as the reported EMS and ED chief complaint, with a final diagnosis of heart failure. In another case, change in behaviour was reported as the reason for transfer in LTC, general malaise was listed as the EMS chief complaint, fever as the ED chief complaint and sepsis as a diagnosis, which may reflect the atypical presentation of sepsis in older adults. Of these types of inconsistencies, the most frequently reported reasons for transfer were shortness of breath (n = 22), sudden change in physical condition (n = 18) and falls (n = 16).

Last, documentation in 79 of 227 transitions (34.5%) was identified by independent coders as unclear*.* In these cases, documentation across care settings was neither clearly clinically related, nor solely the result of insufficient documentation (e.g., LTC = skin changes, EMS = general malaise and ED = seizures). The most common reasons for transfer in these cases were sudden change in physical condition (n = 18) and falls (n = 16).

## Discussion

This is the first analysis that we know of that examines the documentation of staff in LTC, EMS and the ED during the emergency transition process. In this context, staff providing the documentation would have included Registered Nurses and Licenses Practical Nurses in LTC, advanced and primary care paramedics in EMS and Registered Nurses at triage in the ED. Our findings demonstrate that over a third of emergency transitions from LTC to the ED have issues with inconsistent documentation, with implications for all involved HCPs and settings.

Discrepancies in documentation across settings may reasonably reflect disease progression or the various manifestations of certain acute conditions. If a resident’s condition worsens during the transition process, documentation changes should reflect these changes. However, it may also point to a need for a common understanding and language regarding what the primary listed reason for transition should be. For example, even though cough and congestion may be occurring, it may not be the most pressing concern that led to an emergency transition. HCPs seeing cough and congestion as a reason for transfer might be influenced to underdiagnose or undertreat a patient, leading to subsequent harm to vulnerable older adults. Issues with quality and accuracy in the context of written documentation has led to adverse patient events as severe as wrong site surgeries in the past [28]. It is unclear whether these sorts of discrepancies are rooted in differing educational and documentation expectations among various HCPs and care settings. Although HCPs may adhere to norms regarding acceptable documentation, an established common terminology related to clinical reasoning for HCPs within EMS is lacking, which may influence documentation [29]. Differences in reporting lie even within the nursing profession, as documentation can reflect nursing diagnoses or direct nursing interventions indicated for treatments in single settings that may not clearly align with accepted medical diagnoses (i.e., the nursing diagnoses directing nursing interventions in LTC may differ from in the ED, and there could be instances in which neither of the nursing diagnoses directly aligns with the resulting medical diagnosis) [30]. Joint education for staff in LTC, EMS and ED to support a shared understanding and improvement of documentation should be explored in future research, as improved documentation can prevent ambiguity and improve communication among HCPs [31]. Some clinical documentation improvement programs, facilitated by a health information specialist, have been attributed to improved patient outcomes such as decreased length of hospital stay [31]. Furthermore, differences in organizational support for continuing education across care settings and health authorities, as well as differences among documentation mediums or software need to be considered and addressed when developing joint education and training [32, 33].

Documentation inconsistencies, or ambiguous cases, may reflect atypical clinical presentations or disease progression by older adults experiencing changes in their health condition [13, 14, 34]. Vague or inconsistent reporting of symptoms for older adults, particularly among those with impaired cognition or limited ability to communicate on their own behalf, puts these individuals at serious risk of receiving insufficient or inappropriate care [3–5]. Given this understanding, we were surprised by the relatively high number of transitions in which sudden change of condition was reported as a reason for transfer (n = 124). Differences in what or how changes in condition are documented could be due to a myriad of contextual reasons such as documenter role or profession, norms in a particular setting, the components of the documentation forms and how familiar HCPs are with a resident [35, 36]. The reasons for differences in documentation across care settings should be explored in further research. We strongly recommend that education and on-the-job training discourage the use of vague descriptors and support the reporting of specific signs or symptoms of concern, particularly for emergency transitions. Moreover, supporting both enhanced written and established verbal communications (such as Situation-Background-Assessment-Recommendation (SBAR) technique) among all members of LTC staff, beyond those providing report to EMS personnel, is warranted to ensure that subtle changes in resident condition are specified and precisely documented [36].

In this analysis, many transitions with inconsistent documentation were due to disease progression or different manifestations of the same condition. Considering that most residents included in the present study had dementia along with other complex comorbidities (see Table 1), atypical presentations were likely a challenge for HCPs involved in emergency transitions from study LTC settings. Notably, there is a lack of education and learning materials on geriatric assessments for EMS [37]. Specifically, EMS professionals perceive a deficit in education for instances when an older adult patient has difficulty communicating or an altered mental status [38]. There is also a lack of education around responses to atypical presentations in undergraduate nursing education [39]. Calls for learner centred approaches utilizing case studies that highlight the important features of atypical presentations in older adults, including how to identify the proper assessment practices, and what signs and symptoms can occur during atypical presentations (e.g., new onset in delirium or change in behaviour and the absence of symptoms related to pain or fever) are proposed for undergraduate nursing education [39]. Given the need for improved interactions and communication between LTC and ED nursing staff, as well as EMS, holding joint education sessions could serve to enhance knowledge *and* relations among HCPs while adhering to principles of cost-containment [21, 38, 40]. Cultivating these partnerships could also serve as a foundation for future work to develop relevant evidence-informed clinical practice guidelines pertaining to documentation practices across care settings during emergency transitions for older adults.

Insufficient reporting, particularly in LTC, generates inconsistencies through the rest of the transition process [1]. A clear reason for transfer or chief complaint is often not reported consistently within the same document, sometimes in inappropriate sections [41]. The value of using pre-filled electronic health records, with mandatory drop-down sections for the vital resident information related to emergency transition, warrants investigation to improve the quality and completeness of information exchanged between care settings [42].

Consistent documentation typically occurred when falls or shortness of breath were the primary reason for transfer for LTC. However, it is important to note that falls and shortness of breath were also common reasons for transfer within categories of inconsistent documentation (e.g., fall followed by seizures or nausea/vomiting/diarrhea). It may be that when the consequence of a fall is a primary concern, documentation is more intuitively consistent across settings, but when HCPs are more concerned about potential causes of falls (e.g., seizure) external chart review will not be able to identify consistency across settings. Further exploration of clinical reasoning for reporting falls is warranted, and transition situations related to falls among residents of LTC could be used as case examples in continuing education activities. It may also be that, while falls are important medical history, they should not be reported as the sole reason for transfer. Emergency HCPs may be better able to provide care if LTC HCPs document the condition they believe warrants emergency transfer (whether that is the cause or consequence of the fall).

### Strengths & limitations

This study has limitations. We collected data from two cities in Canada. While these two cities differ in size, and represent different health systems in different provinces data collection from more cities would have resulted in greater generalizability of our findings. We did not collect information on the experience of the HCPs involved in each transition, which can influence clinical reasoning and related documentation. If the primary trigger event leading to transfer or chief complaint was documented in other places, such as comment boxes or in the ‘trigger event in the last seven days’, it was not considered for this analysis. We were not able to consider how facility or regional policy, or the documentation form available to staff in each care setting might influence documentation. It is important to acknowledge that inconsistent documentation may also reflect warranted corrections to the reported reason for transfer or chief complaint. In these instances, correcting for ‘momentum’ cognitive bias, in which persons adopt the diagnosis or reporting offered by another HCP, would be appropriate and encouraged practice among HCPs [43]. We did not collect any data related to this to examine but recognize it should be considered when interpreting our findings. However, this is the first and only study that we know of that examines the nature of documentation consistency for reasons for transition among LTC, EMS and the ED for transitions. It provides novel information about documentation across various care settings during the whole transition process. We included all transitions from LTC to the ED, regardless of disposition from the ED. Our research team members have substantive and clinical expertise across all relevant settings, thereby strengthening the directed content analysis conducted for this paper.

## Conclusions

Insufficient and vague reporting can lead to missed care, under or overtreatment, and significantly reduced quality of care throughout the transition process. Our results demonstrate the need for shared, cross-setting continuing education to ensure that documentation is sufficient, supports a geriatric focus and considers differing HCP perspectives around best documentation practices. Improving documentation is critical to improving the delivery of optimal care and to reducing unnecessary transitions in the future, which can stem from a root source of too little information.

## Data Availability

The datasets generated and/or analysed during the current study are not publicly available due to the sensitive nature of patient data and related ethical requirements but are available from the corresponding author on reasonable request.

## Abbreviations

AB: Alberta
BC: British Columbia
CIHR: Canadian Institutes of Health Research
CTAS: Canadian Triage and Acuity Scale
ED: Emergency Department
EMS: Emergency Medical Services
HCP: Healthcare Professionals
LTC: Long-term care
OPTIC: Older Persons’ Transitions in Care
RA: Research Assistant
RN: Registered Nurse
SBAR: Situation-Background-Assessment-Recommendation
SD: Standard Deviation
